# AmotP130 regulates Rho GTPase and decreases breast cancer cell mobility

**DOI:** 10.1111/jcmm.13533

**Published:** 2018-01-29

**Authors:** Zhe‐Ling Chen, Jiao Yang, Yan‐Wei Shen, Shu‐Ting Li, Xin Wang, Meng Lv, Bi‐Yuan Wang, Pan Li, Wen Zhao, Rui‐Yue Qiu, Yu Liu, Pei‐Jun Liu, Jin Yang

**Affiliations:** ^1^ Department of Medical Oncology The First Affiliated Hospital of Xi'an Jiaotong University Xi'an China; ^2^ Department of Oncology Shangluo Central Hospital Shangluo Shaanxi China; ^3^ Department of Biology & Biochemistry University of Houston Houston TX USA; ^4^ Center for Translational Medicine The First Affiliated Hospital of Xian Jiaotong University Xi'an Shaanxi China

**Keywords:** angiomotin, breast cancer, cytoskeleton, invasion, Rho

## Abstract

Angiomotin (Amot) is a newly discovered, multifunctional protein that is involved in cell migration and angiogenesis. However, the role of its isoform, AmotP130, in the regulation of cytoskeleton and metastasis of breast cancer, is unclear. The aim of this study was to investigate the role of AmotP130 in the reorganization of the actin cytoskeleton and the changes of morphology in breast cancer cells through the Rho pathway that influences the invasion and migration of cells. The results suggested that AmotP130 suppressed the invasion ability through remodelling the cytoskeleton of breast cancer cells, including the actin fibre organization and focal adhesion protein turnover. Global transcriptome changes in breast cancer cells following knockdown of AmotP130 identified pathways related with the cytoskeleton and cell motility that involved the Rho GTPase family. From database analyses, changes in the Rho GTPase family of proteins were identified as possible prognostic factors in patients with breast cancer. We have been suggested that AmotP130 suppressed the invasion ability through remodelling of the cytoskeleton of breast cancer cells, involving regulation of the Rho pathway. The cytoskeleton‐related pathway components may provide novel, clinically therapeutic targets for breast cancer treatment.

## 1 INTRODUCTION

Breast cancer is a major cause of mortality in females. In 2017, there were 40 610 breast cancer‐related deaths in US females, second to lung and bronchial cancer.^1^ Cancer metastasis is the main reason for poor survival and the increasing difficulties of treatment.^2^ New therapeutic strategies and novel targets are therefore necessary for the treatment of metastatic breast cancer. At the molecular level, changes in cell polarity are important in the metastatic process that is driven by substantial reorganization of the cytoskeleton.^3^ Some studies have identified new molecules related with these processes, including apoptosis‐linked gene 2 (ALG‐2),^4^ the polo family serine threonine kinase Plk4^5^ and LMO2.^6^ However, knowledge of the cytoskeleton regulators of metastasis is still limited and the concrete mechanism is not clear.

Angiomotin (Amot), a newly discovered molecule, was identified from its ability to bind to angiostatin using a yeast two‐hybrid screening system.^7^ Previous studies have demonstrated that Amot is related to the cell migration, tube formation and angiogenesis promotion.^8^, ^9^ Two isoforms of Amot, AmotP80 and AmoP130 have been identified. Compared with AmotP80, AmotP130 contains an extended N‐terminal domain.^10^ Notably, AmotP130 was reported to associate with F‐actin through a conserved F‐actin‐binding domain bundle, whereas the AmotP80 was not. To some extent, the binding was related with actin bundling and other rearrangements of the cytoskeleton. However, the role of AmotP130 in the regulation of the cytoskeleton and metastasis of breast cancer is unclear.^11^

In vitro studies suggested that cell directional metastasis is composed of a series of processes, including cell protrusion formation,^12^ focal adhesion (FA) establishment,^13^ cell contraction and FA turnover.^14^, ^15^ FAs are composed of different proteins including vinculin, α‐actinin and Arp2/3.^16^ The adhesive structures bridge between the actin cytoskeleton and extra cellular matrix (ECM).^17^ The actin architectures are integrated and produce the force and movement.^18^ Together, a coordinated set of procedures regulates cell migration.

The mechanism whereby AmotP130 affects cell morphology, adhesion and invasion ability through actin filaments organizations is unknown. In this study, we showed that AmotP130 inhibited cell adhesion and invasion ability. The processes could be controlled by the cytoskeleton reorganization and were related to the Rho pathway components.

## 2 MATERIALS AND METHODS

### 2.1 Antibodies and staining

AmotP130 antibody (sc‐166924) was purchased from Santa Cruz Biotechnology (Santa Cruz, CA, USA); FAK (66258‐1‐lg) and glyceraldehyde 3‐phosphate dehydrogenase (GAPDH; HRP‐60004) were purchased from Proteintech (Wuhan, China); P‐FAK (ab81298) was purchased from (Abcam, Cambridge, MA, USA); vinculin, E‐cadherin, N‐cadherin, vimentin, RhoA, Rac1, Cdc42 and ROCK1 were purchased from Cell Signaling Technology (Danvers, MA, USA).

### 2.2 Drugs

Zoledronic acid (ZOL) was kindly provided by Novartis (Shanghai, China). The drug was solubilized and stored at −20°C. It was diluted in medium before use.

### 2.3 Cell culture

The MCF7, T47D, ZR75, BT474, MDA‐MB‐231, MDA‐MB‐468, HCC1806, HCC1187, MDA‐MB‐453 and SKRB3 breast cancer cell lines, and the MCF‐10A and MCF‐12A breast epithelial cell lines were purchased from the American Type Culture Collection (ATCC; Manassas, VA, USA). Cell expansion of MCF‐10A and MCF‐12A cells was performed in Dulbecco's Modified Eagle's Medium (DMEM)/F12 (Invitrogen, Carlsbad, CA, USA) supplemented with 5% (v/v) horse serum (HyClone, Logan, UT, USA), 1% (v/v) penicillin/streptomycin, 0.5 μg/mL hydrocortisone, 10 μg/mL insulin (both from Sigma‐Aldrich, St. Louis, MO, USA) and 20 ng/mL recombinant human epidermal growth factor (Invitrogen). MCF‐7, T47D, ZR75, MDA‐MB‐231, MDA‐MB‐468, HCC1187 and MDA‐MB‐453 breast cancer cell lines were cultured in DMEM supplemented with 10% (v/v) foetal bovine serum (FBS) (both from HyClone). BT474, HCC1806 and SKRB3 cells were cultured in RPMI‐1640 medium supplemented with 10% (v/v) FBS (both from HyClone). All cells were maintained at 37°C in a humidified atmosphere containing 5% CO_2_.

Lentiviral‐mediated knockdown of Amot‐p130 in MCF7 breast cancer cell lines and overexpression in MDA‐MB‐231 cell lines were used. The RNA interference sequences used for AmotP130 knockdowns were: Angiomotin shRNA1, 5′‐CATACACCAGCAAGCCACAGGGAAT‐3′, and Angiomotin shRNA2, 5′‐CAAGAATCCCACAAGTTCCAGTGAA‐3′. Lentivirus vectors were constructed, generated and purified by Hanbio Biotechnology Co., Ltd. (Shanghai, China). The overexpression sequences were found in the GenBank database (NM_001113490.1). Lentivirus vectors were also constructed, generated and purified by GeneChem (Shanghai, China). The methods of lentivirus infection were as previously published.^19^

### 2.4 Western blotting

The lysis buffer and the procedure for membrane and cytosolic protein purification were as previously published.^20^ The cell lysates were sonicated to disrupt cell membranes and release cytosolic proteins. Lysates were ultracentrifuged at 105 × *g* for 1 hour at 4°C. The supernatant collected is the cytosolic fraction, and the resulting precipitate is the membrane fraction. RIPA buffer (50 mmol/L Tris [pH 7.5], 1 mmol/L EDTA, 100 mmol/L NaCl, 2.5 mmol/L sodium orthovanadate, 10 μL/mL protease inhibitor cocktail, 1 mmol/LPMSF, 0.5% NP40 and 0.5% Triton X‐100) were used to obtain the total protein fraction. The BCA assay (Pierce, Rockford, IL, USA) was used to quantify protein concentrations. Equal amounts of the cell lysate protein (30 μg) were subjected to 10% SDS‐PAGE, and the gels were then electroblotted using nitrocellulose membranes (Millipore, Boston, MA, USA). The membranes were then blocked with 5% non‐fat dry milk in Tris‐buffered saline with Tween‐20 for 2 hours and incubated with the indicated primary antibody at 4°C overnight. The bound antibodies were detected with horseradish peroxidase HRP‐conjugated secondary antibodies (1:5000; Cell Signal Technology). The reactive bands were visualized by chemiluminescence with the Luminol reagent (Millipore). GAPDH was used as a loading control.

### 2.5 Immunofluorescent staining

The procedures used for immunofluorescent staining have been previously reported.^19^ Rhodamine phalloidin was used for the primary antibody staining procedure, conducted at room temperature for 30 minutes under dark conditions. The secondary antibody and nuclear staining were the same as other Immunofluorescent staining.^19^

### 2.6 Cell adhesion assay

For cell adhesion assays, 96‐well plates were incubated with Matrigel^®^ (1:8 in phosphate‐buffered saline [PBS]) at 37°C overnight. The excess medium was removed and serum‐free medium (50 μL per well) was further incubation at 37°C, for 30 minutes. Cells were removed and resuspended at 1 × 10^5^ cells/mL. For each cell line, 100 μL of the cell suspension was added into three wells and incubated with Matrigel^®^. Cells (three samples) without Matrigel^®^ served as a control. The plates were incubated at 37°C for 1 hour, and then the excess media and non‐adhered cells were removed. The remaining adhered cells were quantified using the 3‐(4,5‐dimethylthiazol‐2‐yl)‐2,5‐diphenyltetrazolium bromide (MTT; Sigma‐Aldrich) assay. The absorbance was measured at 492 nm using a multifunction microplate reader (POLARstar^®^OPTIMA; BMG Labtech, Ortenberg, Germany). Three independent experiments were performed for each cell line.

### 2.7 Invasion assay

Cell migration chambers consisting of 24‐well plates were used for invasion assays (BD Biosciences, San Jose, CA, USA). Transwell^®^ plates were coated with Matrigel^®^(1:3 dilution with serum‐free DMEM; BD Biosciences), and 600 μL of DMEM containing 10% FBS was added to the lower well. A total of 2 × 10^5^ cells were added without FBS to the Matrigel^®^‐coated 8.0‐μm pore size membranes. After culturing for 24 hours, the cells migrating to the other side were fixed in 95% ethanol for 20 minutes, washed with PBS three times and stained with 0.4% Crystal Violet for 30 minutes. The migrated cells were photographed with a microscope and camera and were then counted in five different fields.

### 2.8 Real‐time polymerase chain reaction analysis

Cells were processed for total RNA isolation according to the manufacturer's protocol (Fast 200 reagent; Pioneer Biotechnology Inc., Shaanxi, China). The cDNA was prepared by the Transcripto, First Strand cDNA Synthesis kit (Roche, Germany) and the procedure followed the manufacturer's instructions. Real‐time quantitative PCR was performed using the Real‐Time PCR Detection System (Bio‐Rad, Hercules, CA, USA) and SYBR Premix Ex Taq^™^ II (Takara). The primer sequences used for amplification were:

ROCK1: forward (F): 5′‐AACATGCTGCTGGATAAATCTGG‐3′, reverse (R): 5′‐ TGTATCACATCGTACCATGCCT‐3′;
Rac1: F: 5′‐GGGAGACGGAGCTGTAGGTAAAAC‐3′, R: 5′‐AGCGCCGAGCACTCCAGGTAT‐3′;
Cdc42: F: 5′‐ATTATGACAGACTACGACCGCT‐3′, R: 5′‐AGTGGTGAGTTATCTCAGGCA‐3′;
PAK3: F: 5′‐CCAGATCACTCCTGAGC‐3′, R: 5′‐CCAGATATCAACTTTCGGACC‐3′;
Sep7: F: 5′‐AGGGCAGCTGACTAAGAGCCC‐3′, R: 5′‐TCATTTGCTCATGGCGCCGCT‐3′.

### 2.9 Live‐cell imaging and time‐lapse movies

Cells were seeded on culture dishes and mounted on the stage of a Leica DM16000 B microscope (Leica, Wetzlar, Germany). Before the recording, two different fields that had the lowest cell density were chosen. Images at the same position were photographed at 20× magnification every 15 minutes for 24 hours. These images were then compiled into time‐lapse movies performed with ImageJ software (Image J, version 1.46r; NIH, USA).

### 2.10 Animal metastases model

All animal experiments are in line with the guidelines of the Institutional Animal Care and Use Committee and approved by the Animal Ethics Committee of Xi'an Jiaotong University. Four‐week‐old non‐obese diabetic severe combined immunodeficiency (NOD‐SCID) female mice were housed in a special pathogen‐free animal facility. For orthotopic xenografts, 2 × 10^6^ cells were injected into the mammary fat pads of both sides. At the end of the sixth week, all mice were killed and the lung tissues of mice were dissected to obtain lung metastases.

### 2.11 Statistical analysis

The SPSS statistical software (SPSS, version 18.0; Chicago, IL, USA) was used for statistical analysis. A value of *P* < .05 was considered as statistically significant. The statistical tests were two‐sided.

## 3 RESULTS

### 3.1 Different cellular morphology and cytoskeletal organization depended on the AmotP130 expression in breast cancer cells

**Figure 1:**
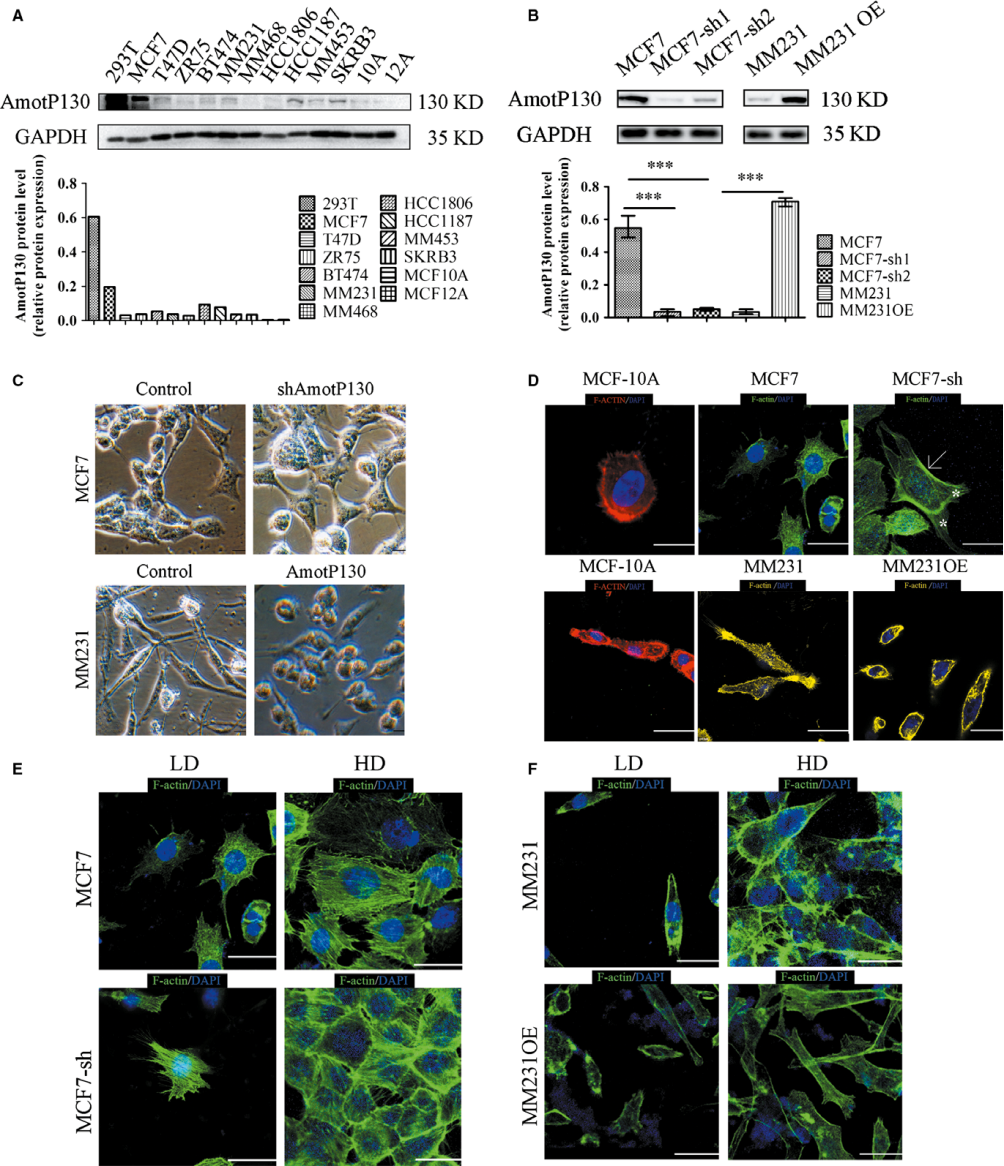
AmotP130 was differential expressed in breast cell lines and correlated with cell morphology and cytoskeleton arrangement. MCF7‐sh, the AmotP130 silenced MCF7 cells; MM231, MDA‐MB‐231 cells; MM231 OE, the AmotP130 overexpressed MDA‐MB‐231 cells; LD, low cell density; HD, high cell density. A, Western blot analysis of AmotP130 expression in positive control cell (293T) and breast cancer cell lines. B, Western blot verification of knockdown and overexpression efficiency of AmotP130 in MCF‐7 and MM231 cells. C, Images of different cell morphology depending on the AmotP130 expression status under 200× field. D, Representative fluorescent images of F‐actin cytoskeleton labelled by phalloidin showed different actin microfilaments arrangement depended on the AmotP130 expression status. E, Representative fluorescent images of F‐actin cytoskeleton labelled by phalloidin in MCF7 and MCF7‐sh cells under LD and HD. F, Representative fluorescent images of F‐actin cytoskeleton labelled by phalloidin in MM231 and MM231OE cells under LD and HD. Scale bar 10 μm. Values were mean ± SD. ***P < .001

The protein expression of AmotP130 in 293T positive control cells (high AmotP130 expression), MCF7, T47D, ZR75, BT474, MDA‐MB‐231, MDA‐MB‐468, HCC1806, HCC1187, MDA‐MB‐453 and SKRB3 breast cancer cell lines, and MCF‐10A and MCF‐12A breast epithelial cell lines were detected by western blotting. The results showed that AmotP130 protein expression highest in the MCF7 breast cell lines. The MDA‐MB‐231cells expressed low levels of AmotP130 (Figure 1A). We established stable AmotP130 knockdown in MCF7 cells (MCF7‐sh1 and MCF7‐sh2) and AmotP130 was overexpressed in MDA‐MB‐231 cells (MM231 OE) using lentiviral transfection. The knockdown and overexpression efficiency was observed for the two knockdown cell lines, we chose MCF7‐sh1 to perform the following experiments (designated as MCF7‐sh). Microscopy was used to observe the morphological changes of the cells (Figure 1C). Knockdown of AmotP130 led to a marked invasive phenotype, as demonstrated by a shuttle or polygonal shape. Apophysis accompanied the irregular appearance in MCF7‐sh cells, whereas the MCF7 cells appeared more cobblestone‐like. The MM231 cells were spindle‐like and had slender pseudopodia. Overexpression of AmotP130 in MM231 cells caused changes in the morphology, and some cells appeared round or oval in shape. These morphological alterations were accompanied by cytoskeleton rearrangements. Immunofluorescence assays were used to analyse the localization and organization of F‐actin fibres in the same cell lines and in the MCF‐10A control cells (Figure 1D). In the MCF‐10A cells, F‐actin organized into shorter and spread filaments throughout the cytoplasm. The cell edges were smooth and less pseudopodia composed the fibres. The distribution of F‐actin was not uniform in MCF7 cellsorMM231 cells, consistent with previous study showing that the prominent F‐actin structures had a diffuse distribution in cancer cell lines with less found in the cytoplasm.^21^ The actin fibres in MCF7‐sh cells were confined to cell borders (indicated by an arrow); the actin fibres organized into bundles (indicated by an asterisk), and the protrusions composed of fibres were distributed at the front and the rear of the cells. The actin fibres at the cell borders seemed less obvious in MM231OE cells than in MM231 cells. Actin filaments in the MM231OE cells showed a spotty and scattered distribution pattern. To explore the cytoskeleton structure at regions of intercellular contacts, cytoskeleton organizations were analysed under low density or high density (Figure 1E,F). At high density, the actin fibres in the MCF7‐sh cells were assembled as contractile bundles in the cytoplasm and were gathered at regions with the cells contacting each other, whereas no aggregated distribution appeared in MCF7 cells. The same phenotype was observed in the MM231 and MM231OE cells.

### 3.2 AmotP130 affected cell adhesion and focal adhesion complexes

**Figure 2:**
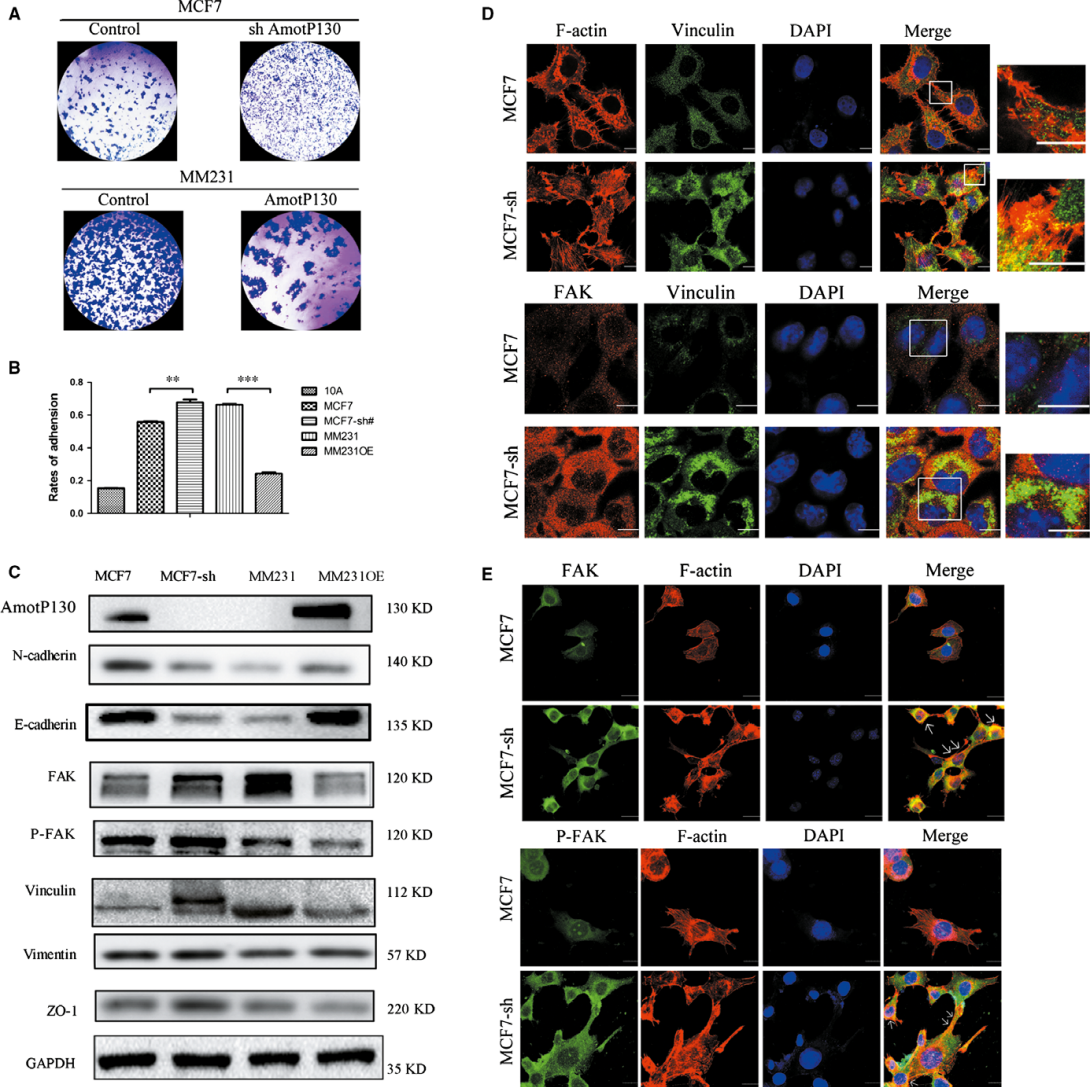
AmotP130 affected cell adhesion and focal adhesion complex. MCF7‐sh, the AmotP130 silenced MCF7 cells; MM231 OE, the AmotP130 overexpressed MDA‐MB‐231 cells. A, Images of MCF7, MCF7‐sh, MM231, MM231OE cells adhered to matrigel were shown. Cells were planted on matrigel for 1 h and adhered cells on the matrigel surface were stained and photographed. B, The number of adhered cells was quantified by MTT assay. The adhesion rates were calculated using the average values of matrigel triplicates compared to the blank triplicates. C, Western blotting detected the changes of adhesion‐related proteins (E‐cadherin, N‐cadherin, Vinculin, FAK, P‐FAK, Vimentin, ZO‐1) in MCF7, MCF7‐sh, MM231, MM231 OE cells. GAPDH was used as an internal control. D, Immunofluorescence assay showed double staining experiments of F‐actin and Vinculin in MCF7 and MCF7‐sh cells (upper panel), and the organization of FAK and Vinculin (down panel). E, Immunofluorescence assay showed the localization of FAK (upper panel) and P‐FAK (down panel) with F‐actin differed in MCF7 and MCF7‐sh cells. The white arrows indicated the colocalization of FAK/P‐FAK with F‐actin in the adhesion sites. Scale bar 10 μm. The values represented the means ± SD. **P < .01, ***P < .001

The results showed that F‐actin assembled in bundles and gathered to the borders of cells after AmotP130 knockdown. The cytoskeletal rearrangement has been reported to be mediated by cell adhesion and focal adhesion kinase (FAK) activation.^22^, ^23^ Thus, it was conceivable that AmotP130 affected the cytoskeleton rearrangement through interfering with the cell adhesion ability and FAK activation. We analysed the cell adhesion ability under various levels of AmotP130 expression (Figure 2A,B) and found that knockdown of AmotP130 increased the adhesion ability, whereas overexpression reduced adhesion. Western blot analyses of adhesion‐related proteins were performed to confirm these results (Figure 2C). The expression of FAK, phosphorylated FAK (P‐FAK) and vinculin was obviously influenced by the AmotP130 expression status. These results indicated that AmotP130 decreased the activity of the FA complex, down‐regulated the adhesion‐related protein expression, and thereby reduced cell adhesion. To determine the effects of AmotP130 on FAs formation, F‐actin organization, FAK, a signal transduction molecule, and vinculin, a membrane anchor protein, were immunostained to observe the adhesion structures before and after AmotP130 knockdown in MCF7 cells. Actin reorganization assays (Figure 2D, upper panel) showed the actin fibres emanating from the FA position where vinculin and F‐actin were colocalized. In control MCF7 cells, the intensity of vinculin staining was weak, and colocalization with FAK was difficult to observe (Figure 2D, lower panel). After AmotP130 knockdown, the expression pattern of vinculin changed and was obviously accumulated at FA sites along with FAK. These data were also verified in AmotP130 overexpressing cell lines. AmotP130 overexpression obviously inhibited FAs formation, and the cytoskeleton gathered at the FAs on cell borders (Figure S1A,B). Statistics analysis showed significant differences in the number of colocalization of FAK and viculin (Figure S2A).

It has been reported that FAK is a core component of adhesion complexes^24^ and plays an essential role in FA turnover.^25^ To explore how this complex might affect the F‐actin arrangements and link the cytoskeleton with the cell aggressive morphology, we used immunostaining to observe the distribution of FAK/P‐FAK and F‐actin, before and after AmotP130 knockdown in MCF7 cells. Compared with MCF7 cells, FAK was detected more abundantly as puncta in the cell edges and FA sites in MCF7‐sh cells. In addition, FAK colocalized more with F‐actin in the adhesion sites (indicated by the arrows, Figure 2E, upper panel). The expression patterns of P‐FAK were similar to that of FAK in the same cell lines (Figure 2E, lower panel). This indicated that low levels of AmotP130 induced the colocalization of FAK and P‐FAK with actin cytoskeleton that gathered into bundles near the cell borders. These results were further verified in AmotP130 overexpressing cell lines (Figure S1C). The colocalization with F‐actin and the aggregate expression at the adhesion sites of FAK/P‐FAK were unclear in MM231OE cells, which had high expression of AmotP130, compared with MM231 cells.

These results indicated the AmotP130 expression inhibited the cell adhesion ability. The FAs were reduced by AmotP130, which were related with the emission of actin cytoskeleton bundles. Furthermore, FAK/P‐FAK expression and its close proximity with F‐actin were impaired.

### 3.3 AmotP130 affected cell motor ability and invasion ability in vivo and in vitro

**Figure 3:**
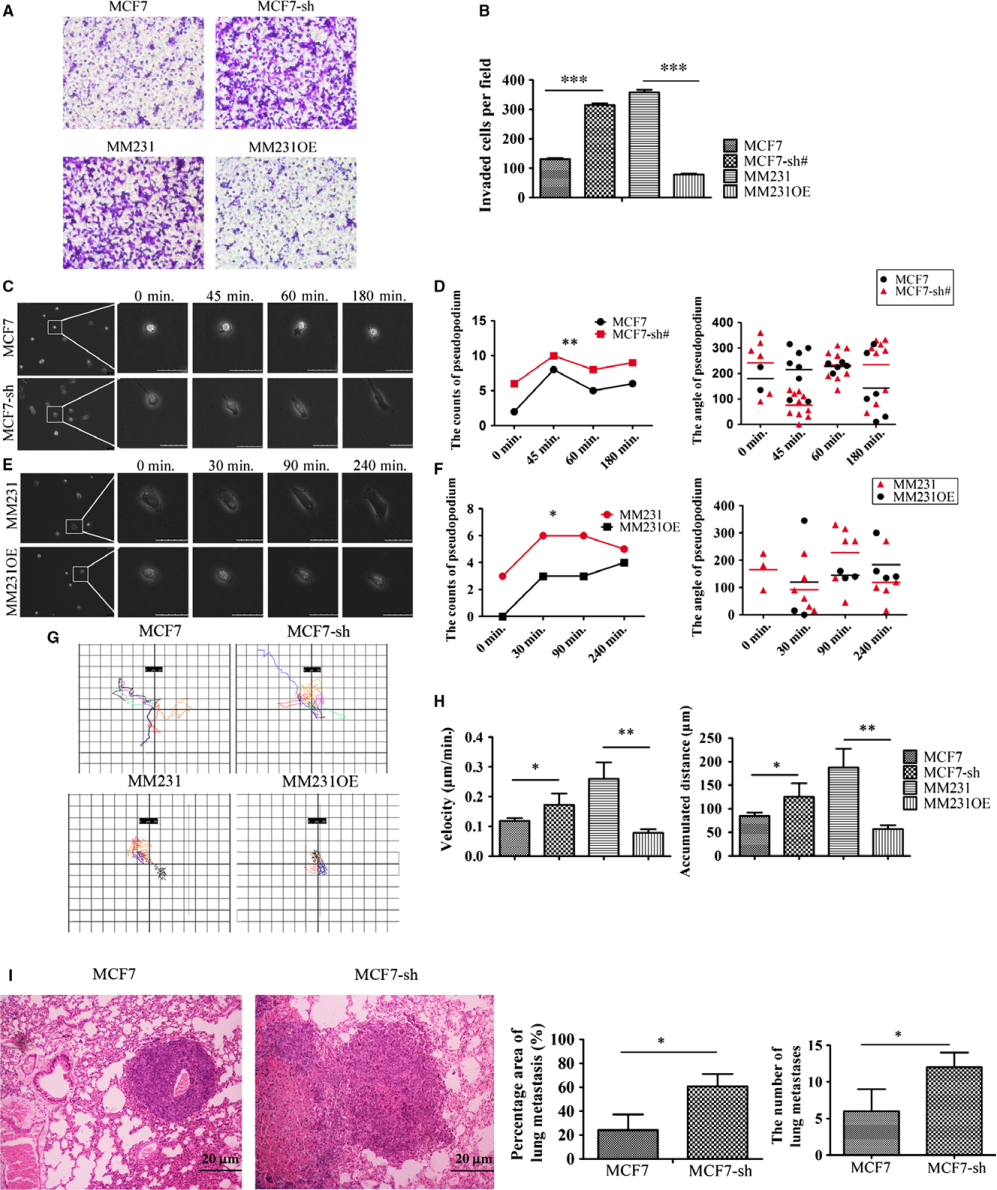
AmotP130 suppressed the invasion and motility ability of breast cancer cells in vivo and in vitro. MCF7‐sh, the AmotP130 silenced MCF7 cells; MM231 OE, the AmotP130 overexpressed MDA‐MB‐231 cells. A, Transwell migration assays in MCF7, MCF7‐sh, MM231 and MM231OE cells. Invading cells were stained and photographed under 200× field. B, Quantity of the mean number of invading cells ±SD from five fields under microscope. C, E, Time‐lapse images of cells were captured every 15 min for 24 h. Scale bar 50 μm. D, F, The number and the angle of pseudopodia for one single cell were quantified. The number of pseudopodia was counted in a cell at the point of time consistent with the time in (C) and (E). The angle of every pseudopodium in the cell showed in (C) and (E) was measured and recorded. G, Cell movement paths were tracked. H, The velocity and the accumulated distance of cell movement were analysed. I, The HE staining of the lung metastasis from MCF7 and MCF7‐sh groups. The statistic analysis was performed to calculate the percentage area and the counts of metastasis sites. The values represented the means ± SD; *P < .05, **P < .01, ***P < .001

Some studies have demonstrated that the changes of cytoskeleton and adhesion ability influenced the invasive potential and motility of cancer cells.^26^, ^27^ Our results suggested that AmotP130 expression status was related with the arrangements of the cytoskeleton. Therefore, we measured the invasion ability and motility of AmotP130 in down‐regulated and overexpressing cells. The results of the Transwell^®^ experiments showed that down‐regulation of AmotP130 induced in vitro invasion of MCF7 cells, and overexpression of AmotP130 significantly reduced invasion of MM231 cells (Figure 3A,B). The formation of pseudopodia and cell motility was then recorded by live‐cell imaging. The images were captured every 15 minutes for 24 hours. When the recording began, the pseudopodia of the MCF7 cells formed after 45 minutes, while that of the MCF7‐sh cells formed within 15 minutes (Figure 3C). The pseudopodia of the MCF7 cells were short and spread throughout the cell and were not related with the cell motor direction. In contrast, the pseudopodia of the MCF7‐sh cells were obviously distributed at the front and the rear of the cells at approximately 1 hour. The front of the cell was flat and the rear was slender. The results showed that AmotP130 down‐regulation increased the number and the angle variance of the pseudopodia (Figure 3D). In addition, the movement pattern of the MCF7‐sh cells was in a directional migration, whereas the motor direction of the MCF7 cells was consistent with the protrusion of cells. MM231 cells showed flat pseudopodia at two sides of the cell at 30 minutes, while the MM231OE cells showed no obvious protrusions until 1.5 hours (Figure 3E). As in the MCF7 and MCF7‐sh cells, the overexpression of AmotP130 in MM231 cells reduced the number and the angle variance of pseudopodia (Figure 3F). The contractibility of the flat pseudopodia forced the movement of MM231 cells; this phenomenon was not obvious in MM231OE cells. We tracked the movement of the cells within a field range (Figure 3G) and found that MCF7 and MM231OE cells appeared to have a smaller range of motion and the movement directions were random. In contrast, the movements of MCF7‐sh and MM231 cells were wider and more directional. We also calculated the velocity of the cell movements and the accumulated migration distances (Figure 3H). The velocity and the accumulated distances of MCF7 and MM231OE cells were less than those of the MCF7‐sh and MM231 cells. These results indicated that AmotP130 decreased the cell invasion and motility. Specifically, AmotP130 suppressed the formation and angle change in the pseudopodia. To illustrate the complete process of pseudopodia formation and the changes in the cells, additional movie files show this process in more detail (Movies [Link], [Link], [Link], [Link]). Next, we performed the metastasis experiments in vitro to verify the effect of AmotP130 played on tumour metastasis ability. We inject the control and MCF7‐sh in NOD‐SCID mice, and the lung metastasis was more obvious in MCF7‐sh group. We performed HE staining and calculated the average area and the counts of lung metastases in two groups, respectively. AmotP130 knocked down obviously increased the counts of metastases. The results indicated that the AmotP130 could suppress the tumour cell metastasis in vitro.

### 3.4 Global gene expression analyses downstream of AmotP130 knockdown revealed several cytoskeleton‐related pathways in MCF7 cells

**Figure 4:**
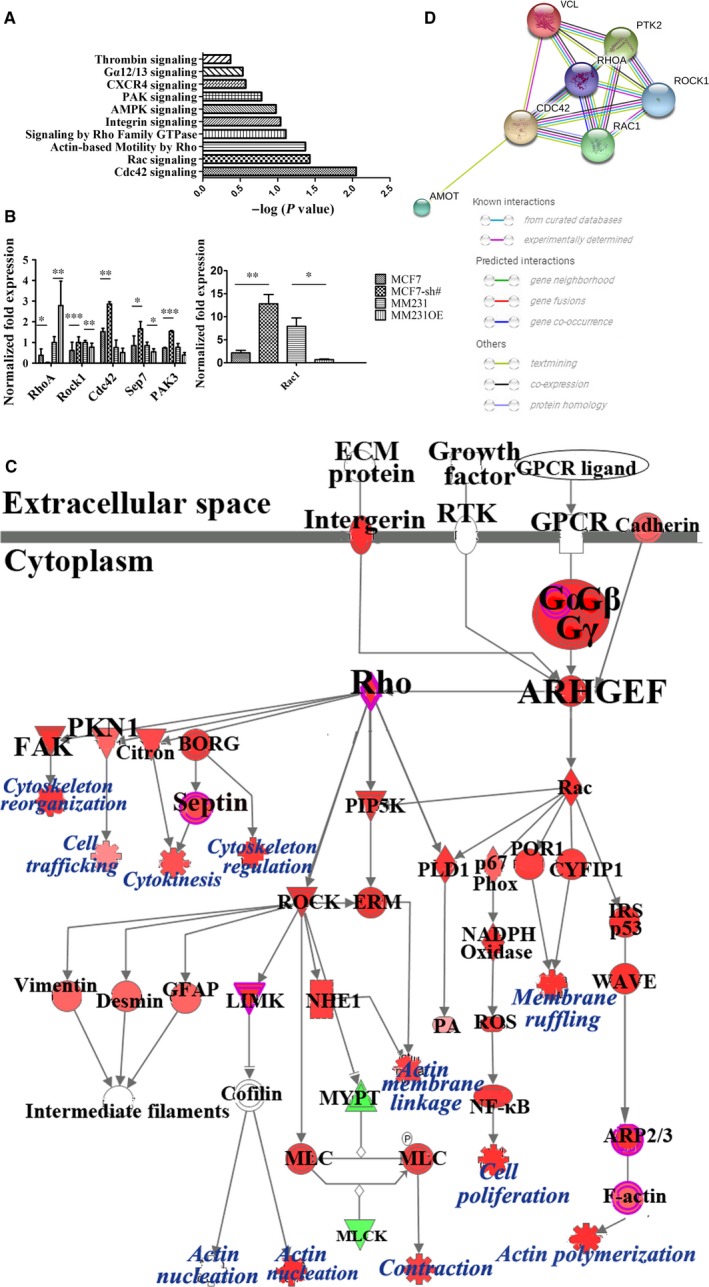
Global gene expression analysis identified the cytoskeleton‐related pathways downstream AmotP130 knocked down in MCF7 cells. A, Functional pathway analysis of the differentially expressed genes based on IPA revealed several cytoskeleton‐related pathways following AmotP130 knockdown. The chosen pathways were ordered by the inverse log of *P* value. B, Confirmation of pathway analysis by qRT‐PCR of key molecules including RhoA, Rac1, Cdc42, PAK3, Sep7 and Rock1 in established AmotP130 knockdown and overexpressed cell lines. C, Predicted signalling by Rho Family GTPases. The predicted pathway showed the experimental data on the signal path of the signal transmission. The signal transduction process of different molecules and the regulation of the differential genes were given. The activation predictions of other genes were predicted by molecular activation prediction. The intensity of the node colour indicated the degree of up‐ (red) or down‐ (green) regulation following AmotP130 knockdown in MCF7 cells. D, The network analysis from the STRING website. The network showed results obtained upon entering proteins including AmotP130, RhoA, Rac1, Cdc42, Rock1, FAK and Vinculin. This was the prediction of protein‐protein association. *P < .05, **P < .01, ***P < .001

To gain further insight into the mechanisms of AmotP130 regulation of cytoskeleton function, we compared the transcriptomes of MCF7 cells and MCF7‐sh cells (AmotP130 knockdown cells). Functional analyses of the genes performed with Ingenuity Pathway Analysis (IPA) revealed that AmotP130 knockdown modulated some pathways related with the cytoskeleton, including Cdc42‐, Rac‐, RhoA‐ and actin‐based motility by Rho signalling (*P* < .05; Figure 4A). An additional file shows the related pathways and molecules in more detail (see Table S1). All the listed pathways were activated when AmotP130 was knocked down. Notably, the pathway analyses showed that Rho GTPase family members played important roles in regulating the cytoskeleton rearrangement and cell motility downstream of AmotP130. Next, we chose the best‐characterized members of the Rho family of GTPases (RhoA, Rac1 and Cdc42^28^), their downstream effector (Rock1), and some components in the canonical pathways (Table S1). Quantitative real‐time PCR was performed to confirm the pathway analysis results (Figure 4B). The mRNA expressions of Sep7 and PAK3 were up‐regulated with AmotP130 knockdown and down‐regulated with AmotP130 overexpression. The key members of the Rho GTPase family (Rac1 and Cdc42) and their effector (Rock1) also had the same expression pattern at the mRNA level. RhoA, however, appeared to be consistent with the AmotP130 expression status and was down‐regulated when AmotP130 was expressed at low levels. The changes were statistically significant in the AmotP130 knockdown cells (MCF7 and MCF7‐sh), but not significant in the AmotP130 overexpressing cells (MM231 and MM231 OE).

The microarray results indicated that the Rho family of GTPases might play a role in regulating the cytoskeleton and cell motility downstream of AmotP130. We next analysed the signalling by Rho GTPases using the IPA, and overlaid it with the microarray data (Figure 4C). The interactome of molecules and the regulation of upstream and downstream components included in this pathway are shown in Figure 4C. The results clearly showed that the regulatory function of the signal pathway included cytoskeletal reorganization and regulation, actin polymerization, and cell contraction.

In addition to the gene level analyses, we searched the STRING database, which consolidates known and predicted protein‐protein associations for a large number of organisms. We analysed the protein networks of AmotP130, RhoA, Cdc42, Rac1, Rock1, FAK (PTK2) and vinculin. The predicted interactions of these molecules showed that AmotP130 may interact with Rho family members, although this interaction was text‐mining, meaning that it was derived from the scientific literature.

### 3.5 Rho inhibition suppressed actin filament polymerization and reduced the invasion ability induced by AmotP130 down‐regulation

**Figure 5:**
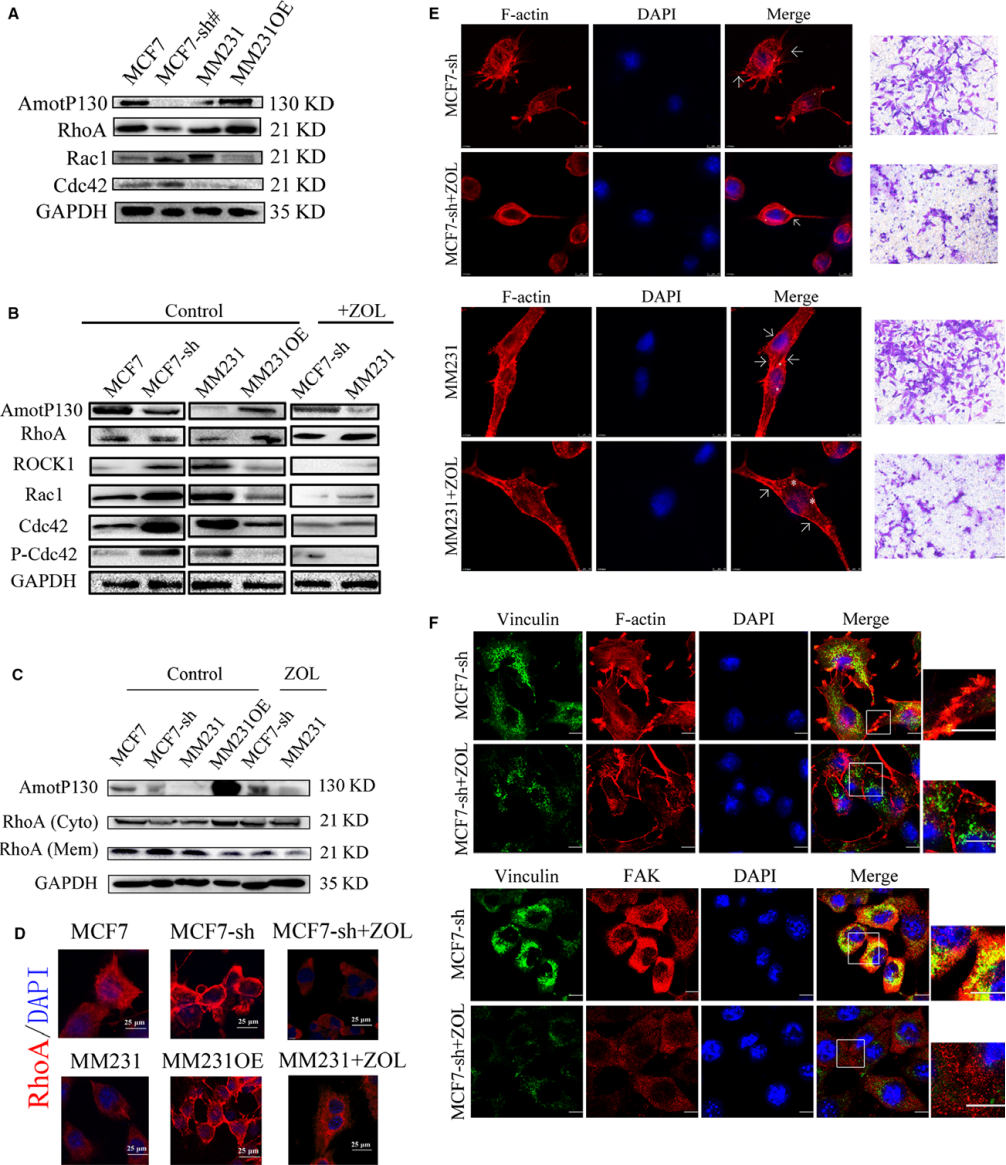
Rho signalling pathway inhibition suppressed actin filaments polymerization and reduced the invasion ability induced by AmotP130 down‐regulation. MCF7‐sh, the AmotP130 silenced MCF7 cells; MM231 OE, the AmotP130 overexpressed MDA‐MB‐231 cells, ZOL, zoledronic acid. A, Western blot analysis of total protein expression of RhoA, Cdc42, Rac1 in MCF7, MCF7‐sh, MM231 and MM231OE cells. B, Western blot analysis of RhoA, Rock1, Cdc42, Rac1, P‐Cdc42/Rac1 total protein expression in cell lines as above. After ZOL 70 μmol L^−1^ treatment for 24 h, these protein were tested again in MCF7‐sh and MM231 cells. C, RhoA membrane and cytosol protein expression were analysed before and after ZOL 70 μmol L^−1^ treatment for 24 h. D, Immunofluorescence assays showed the translocation of RhoA in studied cells before and after ZOL 70 μmol L^−1^ treatment for 24 h. Scale bar 25 μm. E, Immunofluorescence and Transwell migration assays showed the arrangement of F‐actin and invasion ability of MCF7‐sh cells (upper panel) and MM231 cell (down panel) before and after ZOL 70 μmol L^−1^ treatment for 24 h. The asterisk labelled scattered actin and arrow labelled fibres gathered at cell cortex. Invading cells were stained and photographed under 200× field. F, Immunofluorescence assay showed double staining experiments of FAK and vinculin in MCF7‐sh cells (upper panel), and the organization of vinculin and F‐actin (down panel) before and after ZOL 70 μmol L^−1^ treatment for 24 h. Scale bar 10 μm

Because global gene expression and pathway analyses indicated that members of the Rho GTPase family participated in some pathways to regulate the cytoskeleton and invasion ability of breast cancer cells, we used western blots to verify RhoA, Rac1, Cdc42 and ROCK1protein expression in AmotP130 up‐ or down‐regulated cells (Figure 5A). Consistent with the real‐time PCR results, AmotP130 expression inhibited the protein expression levels of Rac1, Cdc42 and ROCK1. These results were combined with the microarray data of pathway analyses and verified that Rho signalling was activated downstream ofAmotP130.

We next inhibited the Rho signalling pathway using ZOL, which was reported to inactivate Rho GTPases.^29^ MCF7‐sh and MM231 cells were treated with 70 μmol L^−1^ ZOL for 24 hours. Western blot analyses showed that ZOL decreased the protein levels of Rac1, Cdc42, P‐Rac1/Cdc42 and ROCK1, but increased RhoA protein levels (Figure 5B).

Previous studies showed that the translocation from cytoplasm to membrane is an early step in RhoA/ROCK signal transduction cascade and the membrane translocation could be an activation form of Rho A.^30^, ^31^ The pathway inhibitor ZOL inhibited membrane RhoA activation.^32^, ^33^ Therefore, the translocation of RhoA to the membrane might be more reliable than the total protein level to indirectly measure the activation of RhoA. We therefore further examined the RhoA protein levels in the cytosol and membrane fractions (Figure 5C). Before treatment with ZOL, RhoA cytoplasmic levels were decreased by AmotP130 knockdown and increased by its overexpression; the membrane levels of RhoA were opposite to that in the cytoplasm. After 24 hours of treatment with ZOL, in MCF7‐sh and MM231 cells, the membrane RhoA protein increased whereas the cytoplasmic level decreased. These results suggested that AmotP130 knockdown activated the Rho‐related pathway, including the phosphorylation of Rac1/Cdc42 and the translocation of RhoA from the cytoplasm to the membrane. This activation could be inhibited by AmotP130 overexpression or RhoA inhibitor. Additional experiments of immunofluorescence assay on the location of RhoA were performed (Figure 5D). The membrane location of RhoA is more obvious after the knockdown of AmotP130 in MCF7 cells. On the other hand, RhoA translocated from membrane to cytoplasm in MM231 cells after the AmotP130 overexpression. The Rho inhibitor ZOL also reversed the cyto‐membrane translocation of RhoA. To determine if the effects of AmotP130 down‐regulation could be reversed by Rho inhibition, we observed the F‐actin organization and cell invasion ability in MCF7‐sh cells (Figure 5E, upper panel) and MM231 cells (Figure 5E, lower panel). After treatment with 70 μmol L^−1^ of ZOL for 24 hours, the stress fibre formation was less than that in untreated cells. This was clearly observed using confocal microscopy; F‐actin appeared scattered in the cytosol as granules (the asterisk labels scattered actin; Figure 5E). At the cell border, less stress fibres formed into bundles and the cell cortex was smoother in the treated cells (arrow labelling the fibres and cell cortex; Figure 5E). The disorganization of the actin cytoskeleton after Rho inhibition was similar to that of the AmotP130 overexpressing MCF7 and MM231OE cells (Figure 1D). The cell invasion ability induced by AmotP130 down‐regulation was reduced by ZOL treatment. Regarding the FA formation, double staining for vinculin and actin revealed reduced vinculin gathered with actin bundles in the cell adhesion regions after Rho inhibition in MCF7‐sh cells (Figure 5F, upper panel). FAK and vinculin staining showed that fewer FAs were found in MCF7‐sh cells treated with the Rho inhibitor (Figure 5F, lower panel). The results indicated that ZOL treatment, which inhibited the Rho signalling pathway, efficiently reversed the effects of knockdown of AmotP130, including the actin fibre polymerization, and increased invasion ability and FA formation. The results further suggested that downstream of the AmotP130, the Rho pathway and its effects on the cytoskeleton were inhibited.

**Figure 6:**
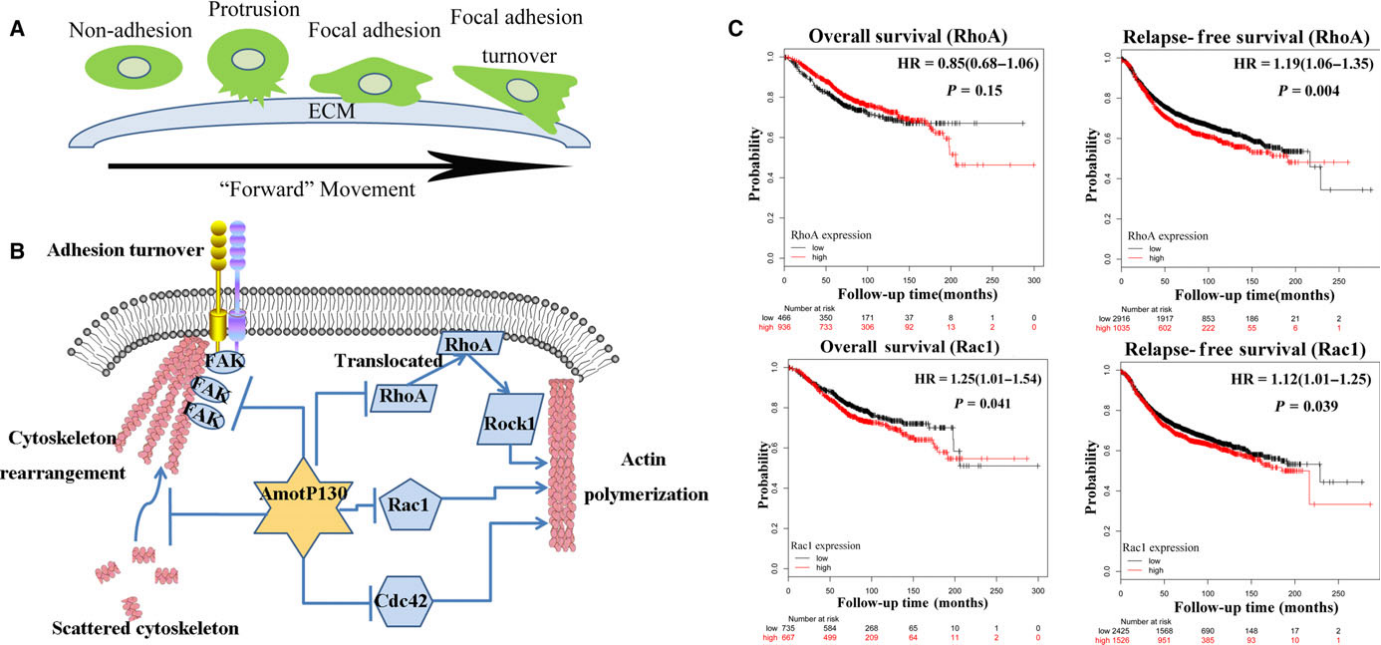
Schematic of the research and the clinical analysis of the cytoskeleton‐related biomarkers. A, Cell forward movement included several steps. First, the cell formatted protrusions from the non‐adhesion state. Then, the cell adhered to the extra cellular matrix (ECM) and the focal adhesion (FA) established. The FAs grasped the ECM and the tension produced by stress fibres contraction made the FAs dissemble and turnover. Finally, the cell contracted and the cell body was squeezed forward. B, From current study, we can infer that AmotP130 regulated the RhoA translocation from membrane to cytoplasm, inhibiting the Rac1/Cdc42 expression as well as its phosphorylation. Also, from our observation, AmotP130 affected the FAK and the focal adhesion formation. As a result, the cytoskeleton rearrangement and actin polymerization were controlled by AmotP130. C, Comparison of overall survival (OS) and relapse‐free survival (RFS) rates by different level of RhoA and Rac1 mRNA expression in patients with breast cancer from integrated database

To extrapolate this study to clinical practice, we searched a single integrated database that was publicly available with supporting follow‐up data.^34^, ^35^ This integrative data analysis tool was used to analyse the prognostic power of the cytoskeletal pathway‐related genes. Figure 6C shows the overall survival (OS) and relapse‐free survival (RFS) information of patients with breast cancer grouped by biomarker expression. Higher expressions of RhoA and Rac1 were correlated with poorer RFS and Rac1 expression, indicating poorer OS. By now, the database could not separate the isoform of Amot, and we have tried to search the database about the survival analysis based on Amot expression without separating the two isoforms. However, the results were negative (Figure S2C).

## 4 DISCUSSION

Amot is an angiostatin‐binding protein that promotes endothelial cell migration.^7^ Our team has published a review about Angiomotin family member previously.^36^ We concluded that in epithelial and endothelial cells, all Amot isoforms were localized to cell tight junctions and played important roles in the apical‐basal polarity and stability of the cytoskeleton. However, the function of Amot was reported differently by different researchers even in the same cell line. We thought that the two isoforms, cell types and experimental conditions, could affect the function of Amot. In tumour cells, the role of AmotP130 is complicated and it has been assigned both oncogenic and tumour suppressor in cancer. But precisely because of the inconsistent reports on AmotP130 and the lacking study in breast cancer, we designed the experiments to explore the function of AmotP130 in breast cancer. In our study, Amot played an important role in promoting breast cancer cell proliferation and invasion.^37^ Recently, studies have indicated that AmotP130 and AmotP80 play different biological functions in vitro. AmotP80 enhanced cell migration and AmotP130 interacted with actin and affected cell shape.^10^, ^38^ In this study, we showed that AmotP130 reduced the adhesion and invasion ability of breast cancer cells. Regarding the mechanism, we found that AmotP130 expression was related with the arrangement and polymerization of F‐actin filaments. Furthermore, microarray analyses showed that Rho GTPase family members were associated with AmotP130, which was reported to regulate the cytoskeleton and cell motility.^28^, ^39^, ^40^, ^41^

Cell motility processes consist of several steps, including protrusion at the leading edge, adherence to the ECM, FA formation and turnover, cell contraction and pushing the cell body forward (Figure 6A). The process is dependent on the dynamic cytoskeleton.^12^, ^13^, ^14^, ^15^, ^42^ As a result, the cells display an aggressive phenotype. In this study, AmotP130 expression was related with a less “aggressive” shape and reduced adhesion ability. This phenomenon was driven by the reorganization of the cytoskeleton as was controlled by AmotP130 expression. It is well known that the actin cytoskeleton is linked with the ECM by FAs^43^ and that cell adhesion is an important component of cell migration.^44^ FA formation and turnover have been reported to be related with the dynamics of the cytoskeleton and regulate cell movement.^45^, ^46^ Here, we identified the proteins FAK and vinculin, which were the core members of the adhesion complex.^24^, ^47^, ^48^ Vinculin is mainly locates at the cell‐cell junctions and at extracellular matrix focal spots, which participates in cellular chemical and chemical signal transduction through the interaction with adhesin proteins, cytoskeletal proteins and cytoskeletal F‐actin. FAK is a non‐receptor tyrosine kinase, which is presented in the cytoplasm. It's activated and then transferred to FAs to catalyze the tyrosine phosphorylation of the target protein on the FAs. We focused on the colocalization of the two proteins to represent the cell FA formation. However, the different signal distribution of two proteins also existed due to the functional differences in the protein. AmotP130 not only decreased the protein levels, but also changed their expression pattern within the cytoskeleton. Therefore, we suggest that AmotP130 is associated with lower invasive potential. AmotP130 reorganized the cytoskeletal proteins, including actin fibres and FAs, thus interfering with cell protrusion, adhesion formation and turnover during the migration process (Figure 6A).

The cell migration observed in cancer cells differs from normal cells and may have mechanistic differences.^49^, ^50^ We speculated in our study that AmotP130 changed the organization of cytoskeleton so that suppressed the cell migration and motility. In videos of living cells, the migration pattern appeared different between cells. AmotP130 knockdown caused the cells to move quickly. The orientation depended on pseudopodium formation and angle change. The effective contraction of the pseudopodia controlled the cell “forward” movement. Therefore, it is reasonable to assume that AmotP130 reduces cell invasion and motility, especially pseudopodium‐dependent movement.

To explore the mechanism behind these phenomenons, we analysed microarray data and identified several functional pathways affected by AmotP130. Notably, the Rho GTPase family members, which were reported to co‐ordinate together and rearrange the actin cytoskeleton,^51^ were also identified in our study. RhoA, Rac1 and Cdc42^52^, ^53^, ^54^ are three prototypical members that co‐ordinate together temporally and spatially. Previous studies suggested that Amot controlled RhoA activity in vitro,^55^ and consequently regulated the cytoskeleton, cell junctions and migration.^56^ We focused on the role of AmotP130 in this regulation. We verified the inhibition of the Rho pathway through Rac1, Cdc42 and P‐Rac1/Cdc42 expression, and RhoA membrane protein localization^12^, ^57^ using ZOL, an inhibitor of the Rho pathway that inhibits the mevalonate pathway.^58^, ^59^ Pathway inhibition reduced the cell invasion ability, scattered actin fibres at the cytoplasm, impaired FAs, and this reversed the effects of AmotP130 down‐regulation. These results implied that AmotP130 regulated the Rho pathway and changed FA and cytoskeleton organization, finally influencing cell invasion and migration. Taking the results of previous articles and our microarray assay together, we pointed out the connection between AmotP130 and Rho GTPase initially (Figure 6B). Using the database, the Rho family members were suggested to have an influence on the prognosis of patients with cancer (Figure 6C). But the analyses about Amot without separating the isoforms were negative (Figure S2C). This information regarding the regulation of cytoskeletal pathways may suggest novel therapeutic targets for cancer treatment. And more clinical researches about the relationship between expression of AmotP130 and the survival of the patients are needed.

## 5 CONCLUSIONS

In conclusion, the experimental results of this study confirmed that AmotP130 was related with suppressed invasion ability through remodelling of the cytoskeleton of breast cancer cells, including actin fibre organization and FA turnover. Notably, the mechanism involved AmotP130 regulation of the Rho pathway. It is tempting to speculate that the cytoskeleton‐related pathway proteins could provide new therapeutic targets for clinical breast cancer diagnosis or treatment.

## CONFLICT OF INTERESTS

The authors confirm that there are no conflict of interests.

## AUTHOR CONTRIBUTIONS

Zhe‐Ling Chen, Jin Yang, Pei‐Jun Liu, Meng Lv and Jiao Yang contributed to the conception and design of the work. Zhe‐Ling Chen, Jiao‐Yang, Shu‐Ting Li performed experiments. Zhe‐Ling Chen, Xin‐Wang, Bi‐Yuan Wang contributed to deal with the data and organize the figures. Rui‐Yue Qiu, Wen Zhao and Yan‐Wei Shen helped with the statistical analysis. Zhe‐Ling Chen carried out the majority of manuscript and Yu Liu, Jin Yang and Pei‐Jun Liu revised it critically. All authors gave final approval of the version to be published.

## DATABASE LINKING

Kaplan‐Meier plotter repository: http://kmplot.com/analysis/index.php?p=service&default=true.

STRING: http://string-db.org/cgi/input.pl?UserId=akT14bIkLsE9amp;sessionId=MXwZuEmBiFIp&input_page_show_search=on.
