# Supplementary figures and images for: AmotP130 regulates Rho GTPase and decreases breast cancer cell mobility
Source: J Cell Mol Med. 2018 Jan 29;22(4):2390–403. doi: 10.1111/jcmm.13533 (PMC5867092; doi:10.1111/jcmm.13533)

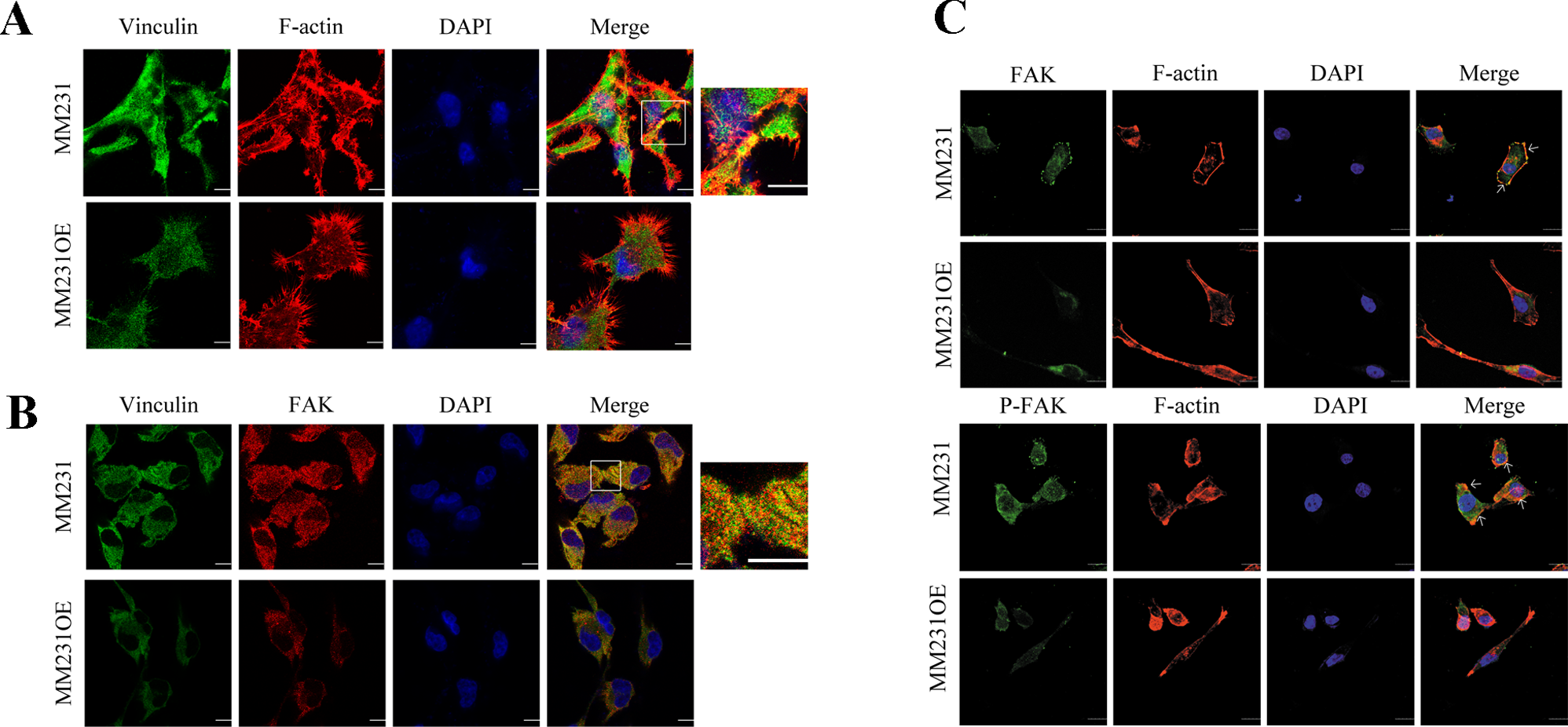

Supplement: Supplementary file 1 [file JCMM-22-2390-s001.tif]

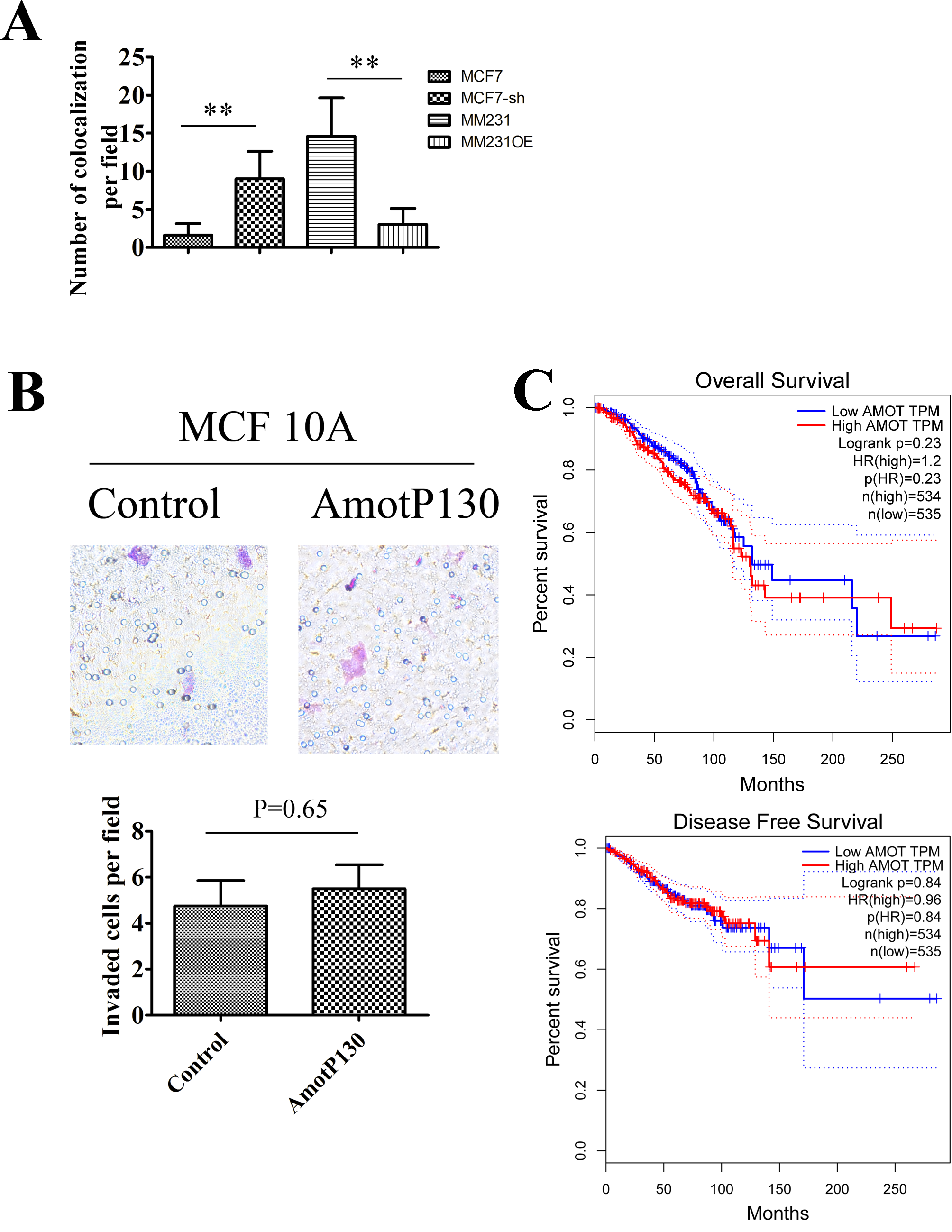

Supplement: Supplementary file 2 [file JCMM-22-2390-s002.tif]
