# Supplementary material for: AmotP130 regulates Rho GTPase and decreases breast cancer cell mobility
Source: J Cell Mol Med. 2018 Jan 29;22(4):2390–403. doi: 10.1111/jcmm.13533 (PMC5867092; doi:10.1111/jcmm.13533)
Supplement: Supplementary file 3 [file JCMM-22-2390-s003.docx]

**TableS1 Canonical Pathway Analysis**

| **Ingenuity Canonical Pathways** | **-log(p-value)** | **Ratio** | **z-score** | **Molecules** |
| --- | --- | --- | --- | --- |
| Cdc42 Signaling | 2.05 | 0.0539 | 0.816 | MAP2K4,IQGAP2,PAK3,HLA-DMA,ARPC5,EXOC5,PPP1CB,LIMK2,HLA-DPA1 |
| Integrin Signaling | 1.04 | 0.0365 | 1.134 | MAP2K4,RND3,PAK3,ACTA2,ARPC5,FGFR2,PPP1CB,PTEN |
| Rac Signaling | 1.43 | 0.0513 | 1.633 | MAP2K4,IQGAP2,PAK3,ARPC5,FGFR2,LIMK2 |
| Regulation of Actin-based Motility by Rho | 1.37 | 0.0549 | 0.447 | RND3,PAK3,ACTA2,ARPC5,PPP1CB |
| Signaling by Rho Family GTPases | 1.11 | 0.0364 | 1.414 | MAP2K4,GNAI2,RND3,PAK3,ACTA2,ARPC5,SEPT7,FGFR2,LIMK2 |

*Here listed the specific results of Ingenuity Pathway Analysis (IPA). The studied pathways and the related molecules also listed out.*
